# Preliminary Analysis of Excess Mortality in India During the COVID-19 Pandemic

**DOI:** 10.4269/ajtmh.21-0864

**Published:** 2022-04-04

**Authors:** Christopher T. Leffler, Joseph D. Lykins V, Saurav Das, Edward Yang, Sneha Konda

**Affiliations:** ^1^ Department of Ophthalmology, Virginia Commonwealth University, Richmond, Virginia;; ^2^ Department of Ophthalmology, Hunter Holmes McGuire VA Medical Center, Richmond, Virginia;; ^3^ Department of Internal Medicine, Virginia Commonwealth University, Richmond, Virginia;; ^4^ Department of Emergency Medicine, Virginia Commonwealth University, Richmond, Virginia;; ^5^ Independent Investigative Journalist, Pondicherry, India;; ^6^ School of Medicine, Virginia Commonwealth University, Richmond, Virginia

## Abstract

We studied all-cause mortality during the COVID-19 pandemic in 19 Indian states (population 1.27 billion). Excess mortality was calculated by comparison with years 2015 to 2019. The known COVID-19 deaths reported for a state were assumed to be accurate, unless excess mortality data suggested a higher toll. Data from one state were excluded due to anomalies. In several regions, fewer deaths were reported in 2020 than expected. Areas in Andhra Pradesh, Delhi, Haryana, Karnataka, Madhya Pradesh, Tamil Nadu, and West Bengal saw spikes in mortality in Spring 2021. The pandemic-related mortality through August 31, 2021, in 18 Indian states was estimated to be 198.7 per 100,000 population (range 146.1–263.8 per 100,000). If these rates apply nationally, then 2.69 million people (range 1.98 to 3.57 million) may have perished in India as a result of the pandemic by August 31, 2021.

## INTRODUCTION

Because SARS CoV-2 testing and death registrations are often incomplete, the full impact of the COVID-19 pandemic is unknown in many regions.^1^ Excess mortality, defined as the increase in all-cause mortality relative to the expected mortality, is assumed to result from infection with the SARS CoV-2 virus, or from the indirect effects of health system overload or social responses to the pandemic.^2^^,^^3^ We sought to integrate published mortality figures from Indian regions to estimate the national pandemic impact.

## METHODS

We used mortality figures published by regional governments and Indian journalists, often from Right-to-Information (RTI) requests (Supplemental References and Supplemental Tables 1 and 2). Most data were archived by the Local Mortality project^2^^,^^4^ or the Development Data Laboratory.^5^ These were supplemented by government hospital data, funeral counts, and handwritten death registers. The Methods are detailed further in the Supplemental Appendix and were approved by the Virginia Commonwealth University Office of Research Subjects Protection.

We acquired mortality data from 19 states (Table 1, Supplemental Table 1). Some smaller states were excluded because relevant mortality data are unavailable. The baseline Uttar Pradesh data contained districts with zero deaths for numerous months and were therefore excluded from the top-line model.

**Table 1:** Reported and estimated COVID-19 deaths in India, 2020 through August 2021

State	Population	Year	Reported deaths/100K	Estimated COVID-19 deaths	
				Deaths/100K	Number
Andhra Pradesh	53,903,393	2020	13.179	121.501	65,493
		2021	12.493	321.772	173,446
Assam	35,607,039	2020	2.929	42.819	15,246
		2021	12.952	–	–
Bihar	124,799,930	2020	1.116	107.567	134,244
		2021	6.619	101.272	126,388
Chandigarh	11,584,730	2020	2.728	2.728	316
		2021	4.290	4.290	813
Delhi	18,710,920	2020	56.240	56.240	10,523
		2021	77.805	230.342	43,099
Gujarat	63,872,400	2020	6.735	65.466	41,815
		2021	9.048	50.688	32,376
Haryana	28,204,692	2020	10.278	39.311	11,087
		2021	24.024	145.458	41,026
Himachal Pradesh	7,451,955	2020	12.493	12.493	931
		2021	35.749	39.930	2,976
Karnataka	67,562,700	2020	17.881	47.896	32,360
		2021	37.316	406.940	274,939
Kerala	35,699,440	2020	8.521	8.521	3,042
		2021	49.387	49.387	17,631
Madhya Pradesh	85,358,970	2020	4.212	4.212	3,595
		2021	8.108	129.304	110,373
Maharashtra	123,144,200	2020	40.167	137.018	168,730
		2021	71.255	86.772	106,854
Odisha	46,356,334	2020	4.036	43.220	20,035
		2021	13.008	125.631	58,238
Punjab	30,141,373	2020	17.687	17.687	5,331
		2021	36.634	120.255	36,247
Rajasthan	81,032,689	2020	3.318	8.181	6,629
		2021	7.731	39.874	32,311
Tamil Nadu	77,841,270	2020	15.556	74.976	58,362
		2021	29.278	250.885	195,292
Telangana	39,362,732	2020	3.915	88.157	34,701
		2021	5.922	71.768	28,250
Uttar Pradesh	237,882,725	2020	3.511	3.511	8,352
		2021	6.082	20.371	48,458
West Bengal	99,609,300	2020	9.721	49.855	49,660
		2021	8.785	56.915	56,692
Total, except Uttar Prad.	1,030,244,067	2020	12.610	64.266	662,100
	994,637,028	2021	24.128	134.416	1,336,951
Total	1,268,126,792	2020	10.903	52.869	670,452
	1,232,519,753	2021	20.645	112.405	1,385,409

Reported COVID-19 deaths are taken from the Johns Hopkins dataset.^6^

Mortality attributed to COVID-19 has been tabulated by the Johns Hopkins University Center for Systems Science and Engineering (CSSE, Supplemental Table 3).^6^ The CSSE obtains data from governmental health authorities^7^ and links to the webpage for the Health Ministry of India.^8^ Indian health authorities were reportedly not attributing deaths to COVID-19 if the patient had comorbidities or SARS-CoV-2 tests were not performed.^9^ We assumed reported COVID-19 mortality reflected the annual pandemic-related mortality in a state, unless excess mortality suggested a higher toll.

For some states, several data sources were available, and we presented the median, lowest, and highest estimates of per capita excess mortality.

We calculated regional excess mortality by comparing the mortality for 2020 or 2021 with the value expected based on linear regression over the years 2015 to 2019.^2^ The Indian government has estimated registration completeness by dividing the death registrations by the state mortality from the Sample Registration System, an annual survey (Supplemental Table 2).^10^ We adjusted for registration completeness as outlined in the appendix.

The national mortality rate was estimated by summing the pandemic-related deaths for the states analyzed and dividing by the population. Indian state populations were taken from the Johns Hopkins dataset.^6^ Raw data are presented in Supplemental Table 2.

To graph mortality timing, mortality rates (deaths divided by population) were calculated for 14 states with monthly data from January 2019 through May 2021. After May, per capita mortality was calculated from five states.

## RESULTS

We studied 19 states, with a combined population of 1.27 billion (Table 1, Supplemental Table 1). Reported per capita COVID-19 mortality (per 100,000) was low in the 19 states studied: 10.9 in 2020 and 20.4 in 2021 as of August 31, 2021 (Supplemental Table 3).

**Figure 1.**
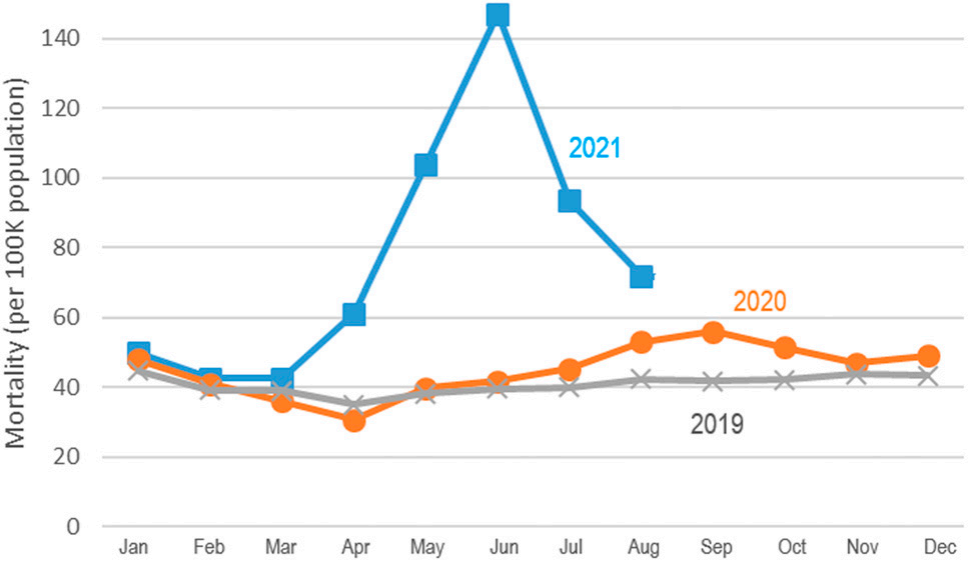
Per capita all-cause mortality in India by month, 2019 to 2021, based on 13 states and two union territories, as described in the Methods section. This figure appears in color at www.ajtmh.org.

In 2020, there was a slight increase in all-cause mortality from August through October compared with 2019 (Figure 1). The most prominent rise in all-cause mortality was evident by April and May of 2021 (Figure 1). After May 2021, available data suggest that mortality registrations continued to rise through June but began to wane thereafter (Figure 1).

For six states, the excess mortality based on registered deaths was actually negative for 2020 (Supplemental Table 1). At the other extreme, the per capita excess mortality (per 100,000) in 2020 was 137.0 in Mumbai (in Maharashtra) and 121.5 in Andhra Pradesh (Supplemental Table 1). For 2020, intermediate levels of excess mortality (per 100,000) were seen for Kolkata in West Bengal (46.1), Chennai in Tamil Nadu (83.5), and Hyderabad in Telangana (88.2) (Supplemental Table 1).

For 2021, a prominent peak in all-cause mortality was seen for March through June for Chennai in Tamil Nadu, Kolkata in West Bengal, Delhi, Madhya Pradesh, Haryana, Punjab, and Andhra Pradesh (Supplemental Table 1). Data available by August 31, 2021, suggested per capita excess mortality (per 100,000) of at least 64.5 for Kolkata, 120.3 for Punjab, 145.5 in Haryana, 212.2 for Madhya Pradesh, 230.3 for Delhi, 265.0 for Tamil Nadu, and 321.8 for Andhra Pradesh (Supplemental Table 1).

We present median values (Table 1) and ranges (Table 2, Supplemental Table 4) for pandemic-related mortality estimates. For most states, the excess mortality exceeded the reported COVID-19 and was therefore taken as the pandemic-related mortality, although there were exceptions (Table 1).

**Table 2:** Estimated COVID-19 mortality in India by August 31, 2021

	Estimated COVID-19 mortality (per 100,000)		COVID-19 deaths (estimated)	
	Median	Range	Median	Range
2020	64.266	47.496–79.341	869,289	642,451–1,073,200
2021	134.416	98.632–184.443	1,818,168	1,334,138–2,494,854
Both years	198.682	146.128–263.784	2,687,457	1,976,589–3,568,054

Assumes population of India of 1,352,642,280. Per capita excess mortality based on all states analyzed except Uttar Pradesh.

In the primary model, data from Uttar Pradesh were excluded because of identified anomalies. For 2020, the pandemic-related per capita mortality (per 100,000) for 18 states with a population of 1.03 billion was estimated to be 64.3 (range 47.5–79.3, Tables 1 and 2). For 2021, through August 31, the pandemic-related mortality (per 100,000) for 17 states with a population of 995 million was estimated to be 134.4 (range 98.6–184.4, Tables 1 and 2). Therefore, we estimate the pandemic-related mortality (per 100,000) to be 198.7 (range 146.1–263.8) for the entire pandemic (through August 31, 2021, Tables 1 and 2). Assuming a population of India of 1,352,642,280, these rates correspond with 2.69 million people (range 1.98–3.57 million) perishing during the pandemic in India from COVID-19 by August 31, 2021.

The estimated COVID-19 mortality can also be expressed as a fraction of the baseline mortality, taken from the 2019 national vital statistics reports (Supplemental Table 4). The estimated COVID-19 mortality represented an increase over the baseline annual mortality of 10.70% (range 7.91–13.21%) in 2020 and 22.40% (range 16.44–30.73%) in 2021 (as of August 31). Pandemic-related deaths in the final 4 months of 2021 will increase these values.

If the data from Uttar Pradesh are included, then the estimated per capita pandemic-related mortality (per 100,000) was 165.3 through August 31, 2021 (range 122.8–217.9, Table 1 and in Supplemental Table 4), corresponding to a mortality from COVID-19 of 2.24 million people (range 1.66–2.95 million) during the pandemic in India.

## DISCUSSION

This analysis of excess mortality found that 2.69 million people (range 1.98–3.57 million) may have perished from COVID-19 in India as of August 31, 2021.

Data from Uttar Pradesh contained anomalies and had an estimated excess mortality lower than other regions, in part because Uttar Pradesh data from after April 2021 were unavailable. However, if the Uttar Pradesh data are included, the estimated pandemic-related mortality in India through August 31, 2021, was 2.24 million (range 1.66–2.95 million).

This mortality level is well above the reported COVID-19 mortality of 438,560 in India as of August 31, 2021.^6^ The Institute for Health Metrics and Evaluation (IHME) at the University of Washington estimated that excess mortality in India was 1.23 million persons on August 31, 2021.^11^^,^^12^

COVID-19 patients who likely would have died during the study period due to their comorbidities even if they had not been infected should not result in excess deaths.

Our analysis used the COVID-19 mortality reported by Johns Hopkins University, unless the excess mortality data suggested a higher pandemic-related toll. Our method of determining the expected baseline by making a projection using linear regression 1 year (for 2020) or 2 years (for 2021) into the future was conservative. Our definition of the baseline by linear regression was used previously in the World Mortality Dataset.^2^

Other groups have estimated Indian pandemic-related mortality to be 4.9 million as of April 2021 based on a household survey^13^; 2.2 million by late May 2021 based on well-defined populations^14^; and, by June 2021, to be 3.2 million^15^ to 3.4 million^13^ based on excess mortality, 2.7 million based on a governmental hospital informatics system,^15^ 3.1 to 3.4 million based on a telephone survey,^15^ and 4.0 million based on seroprevalence.^13^

Our analysis has limitations. The data are incomplete for some regions and times, which might lead to underestimation of mortality. Death registrations may be delayed. Data from regional government websites, central government compilations, and RTI requests are not in complete agreement (Supplemental Table 2). Early data may contain errors. Mortality can be underestimated because of incomplete registration, health system underreporting, and data management errors. Health system overload, delays in patients seeking unrelated healthcare,^2^^,^^3^^,^^16^^,^^17^ and social changes such as lockdowns may produce excess mortality. Excess mortality might also be observed due to other diseases, war, or environmental factors. Mortality deficits, as observed in Australia and New Zealand,^2^ may result from fewer injuries.

## Supplemental Material


Supplemental materials
