# Supplementary material for: Preliminary Analysis of Excess Mortality in India During the COVID-19 Pandemic
Source: Am J Trop Med Hyg. 2022 Apr 4;106(5):1507–10. doi: 10.4269/ajtmh.21-0864 (PMC9128677; doi:10.4269/ajtmh.21-0864)
Supplement: Supplementary file 1 [file tpmd210864.SD1.pdf]

Supplemental Appendix

Supplement to Methods.

This supplemental section elaborates on the methods with greater specificity.

Sources of All-Cause Mortality Data.

We used the publicly available mortality figures published by regional governments and by data journalists in India, often obtained from Right-to-Information (RTI) requests (Supplemental References, Tables S1, S2). Much of these data are stored on the websites maintained by the Local Mortality project of Ariel Karlinsky (Karlinsky 2020, Karlinsky 2021), or the Development Data Lab (2021). These were supplemented by data from government hospitals, funeral counts, and handwritten death registers when available, as detailed below.

We acquired mortality data from 19 states (or union territories) with 1.27 billion population, either for the entire state, or for large cities or districts within the state: Andhra Pradesh, Assam, Bihar, Chandigarh, Delhi, Gujarat, Haryana, Himachal Pradesh, Karnataka, Kerala, Madhya Pradesh, Maharashtra, Odisha, Punjab, Rajasthan, Tamil Nadu, Telangana, Uttar Pradesh, and West Bengal (Tables 1, S1). As a convenient shorthand, we refer to these 19 administrative regions as “states”, even though two of them (Chandigarh and Delhi) are actually union territories. The inclusion of a state or large city in the analysis was based on whether reliable mortality data were available. Some smaller states were not included because governments, reporters, nonprofits and academicians have not yet published relevant mortality data for the pandemic period.

All of these publicly available regional-level mortality data contain no individually-identifiable information. The study was approved by the Office of Research Subjects Protection of Virginia Commonwealth University.

For Chhattisgarh, mortality data from the online portal of the state civil registration system have been released (Data Development Lab), and we included these data in the appendix. However, as the baseline data for this online portal appear to be only about one tenth complete, as compared with the national vital statistics registry, we deemed the Chhattisgarh data too unreliable to include in the model.

For Gujarat, journalists tabulated deaths from March 2020 through April 2021 for 68 of the 170 municipalities, which comprised 6.01% of the state’s population, from the bound handwritten official municipal death registers (Table S2, Supplemental References). For 3 of these Gujarat municipalities (Chorvad, Idar, and Khedabrahma), death register data were available from May 1 to June 10, 2021.

For the urban portions of 25 districts in Madhya Pradesh, the numbers of funerals in April 2021 have been tabulated (Supplemental References).

For Uttar Pradesh, the raw mortality data obtained from a Right-to-Information request contained anomalies, such as multiple districts with zero deaths for numerous months. Therefore, the Uttar Pradesh data were analyzed, but were not included in the top-line model.

Reported Covid-19 Mortality.

Mortality attributed to Covid-19 has been tabulated by the Johns Hopkins University Center for Systems Science and Engineering (CSSE, Table S3, Johns Hopkins University 2021). The CSSE obtains Covid mortality data from the governmental health authorities of the respective countries (Dong 2020). In the case of India, the CSSE links to the Covid-19 webpage for the Government of India Health Ministry (2021). According to reports, local physicians and health authorities in India were in some cases not reporting deaths as caused by Covid-19 if SarsCov-2 tests were not performed or if the patient had contributing comorbidities (Prasad 2021).

Analysis of Mortality.

We assumed that the CSSE figures for Covid-19 mortality accurately reflected the pandemic-related mortality in a given state for each year of the pandemic (2020 and 2021), unless the excess mortality data suggested a higher toll.

For some states, several data sources were available, which permitted the calculation of multiple estimates of per-capita excess mortality. In this case, we presented both the median estimate and the lowest and highest estimates available.

For Gujarat and the urban portions of 25 districts in Madhya Pradesh, mortality data from entire year(s) before 2020 were available (Government of India. Office of the Registrar General 2019). Therefore, to estimate excess mortality for portions of 2021 for these regions, it was necessary to assume that mortality was evenly distributed throughout the year.

For the analysis of data from 68 municipalities in Gujarat, the per-capita mortality for 2021 was estimated by summing the mortality rate through April 2021 with that from available municipalities for May 1 through June 10 (Table S2).

We calculated excess mortality in a region by comparing the mortality for a given time period in 2020 or 2021 with the value expected based on the years 2015 to 2019 (Karlinsky 2020). If data from more than one year before 2019 were available, the expected value was calculated by creating a trend line for mortality by linear regression for the years 2015 to 2019, and carrying this trend one year (for 2020) or two years (for 2021) into the future. Carrying the trend line two years into the future for 2021 yielded conservative estimates of excess deaths.

For some states, reported mortality from the state government websites or RTI requests was only available for 2018 and 2019, which was too short a period to

generate a robust trend line. Moreover, the numbers of deaths from the state sources did not match the central government figures exactly, because the state information systems did not capture all of the registered deaths. In these cases, the vital statistics reports for India were used to generate a trend line for expected deaths, using the data from 2015 to 2019. The expected number of deaths was scaled up or down by multiplying the 2015 to 2019 trend line by the ratio of deaths in the state and federal systems for 2018 and 2019. For instance, if the state website average mortality for 2018 and 2019 was 97% of the figures for 2018 and 2019 in the federal reports, the trend line was multiplied by 0.97. This method was used to scale the trend line for Delhi, Bengaluru, Mumbai, Nagpur, Ahmedabad (for 2020), Madhya Pradesh, Tamil Nadu, for 6 city hospitals in Tamil Nadu, and for Madurai district.

Completeness of death registrations for each state has been estimated by the Indian government in its vital statistics reports by dividing the number of registrations by the state mortality estimated from the Sample Registration System, an annual survey (Table S2, Government of India. Office of the Registrar General 2019). For years in which the completeness was less than 100%, the total number of deaths for each year 2015 to 2019 was determined by dividing the death registrations by the completeness fraction. Unlike the trend line for unadjusted death registrations, the trend line for the adjusted registrations did decrease over time for some states. In order to ensure the estimated excess deaths were conservative, the expected deaths for 2020 and 2021 were the maximum of the 2019 value and the value predicted by the trend line. The completeness fraction for 2020 and 2021 was extrapolated by linear regression from the 2015 to 2019 completeness fraction. Once again, in order to be conservative, the maximum of the 2019 value and the linear extrapolation was used.

The national mortality rate was estimated by summing the estimated pandemic-related deaths for the states analyzed and then dividing by the population of these states. The population size of Indian states was taken from the Hopkins mortality dataset (Johns Hopkins University 2021). Raw data are tabulated in the appendix (Table S2).

Graphical analysis of mortality.

In order to present the timing of the mortality graphically, the per-capita mortality rates (total monthly deaths divided by total population) were calculated for the states for which monthly data were available from Jan 2019 through May 2021 (Andhra Pradesh, Bihar, Chandigarh, Delhi, Haryana, Himachal Pradesh, Karnataka, Kerala, Madhya Pradesh, Maharashtra, Punjab, Rajasthan, Tamil Nadu, and West Bengal). For subsequent months, the per-capita mortality was calculated from states with data available (Punjab through June 2021, Andhra Pradesh through July 2021, Karnataka and Tamil Nadu through August 2021, and Odisha in July and August 2021). For the purposes of generating this figure, the Odisha cumulative annual mortality for August 1, 2021 was estimated by linear interpolation from the July 1 and August 8 values. This

graphical analysis was completely separate from the tabulated estimate of total mortality in India.

Acknowledgments.

The authors would like to acknowledge the data journalists, academicians, and nonprofits in India who have brought these data to light, such as Rukmini S. (@Rukmini), Chinmay Tumbe (@ChinmayTumbe), Vignesh Radhakrishnan (@VigneshJourno), Srinivasan Ramani (@vrsrini), Deepak Patel (@deepakpatel_91), Murad Banaji (@muradbanaji), Sumitra Debroy (@debroysumitra), Dhanya Rajendran (@dhanyarajendran), Shiba Kurian (@shiba_kurian), and Arappor Iyakkam. In addition, we acknowledge the important work of Ariel Karlinsky and colleagues, and the Development Data Lab for preparing many of the mortality databases we used. However, all errors in the paper are the responsibility of the authors.

Supplemental Table S1. Excess mortality in India, January 2020-August 2021.

State	Region.	Population	Excess deaths		Excess mortality / 100K		Final 2021 Date
			2020	Jan-Aug 2021	2020	Jan-Aug 2021	
Andhra Pradesh		53,903,393	65,493	173,446	121.501	321.772	7/31
Assam		35,607,039	15,246	--	42,819	--	--
Bihar		124,799,930	134,244	126,388	107.567	101.272	5/31
Chandigarh		11,584,730	-8,238	-2,707	-71.114	-23.368	5/31
Delhi		18,710,920	1,202	43,099	6.421	230.342	6/30
Gujarat	Entire state	63,872,400	-70,333	32,376	-110.115	50.688	5/10
	Ahmedabad	8,059,000	5,276	2,935	65.466	36.417	5/10
	68 cities	3,838,730	7,004	9,908	182.464	258.094	4/30
	3 cities	83,900	--	145	--	172.295	6/10
	68 cities	3,838,730	--	--	--	430.389	6/10
Haryana		28,204,692	11,087	41,026	39.311	145.458	5/31
Himachal Pradesh		7,451,955	291	2,976	3.902	39.930	5/31
Karnataka	Entire state	67,562,700	12,627	111,876	18.689	165.588	8/30
	Bengaluru	8,443,675	6,510	54,740	77.103	648.291	6/15
Kerala	Entire state	35,699,440	-27,367	6,484	-76.659	18.163	5/31
Madhya Pradesh	Entire state	85,358,970	-28,598	181,159	-33.503	212.232	5/31
	25 districts	25,412,510	--	11,785	--	46.376	4/30
Maharashtra	State less 6 districts	102,598,050	103,152	104,946	100.540	102.288	5/31
	Mumbai city	12,875,213	17,641	6,467	137.018	50.227	5/31
	Nagpur city	2,405,000	4,144	--	172.308	--	
Odisha		46,356,334	20,035	58,238	43.220	125.631	8/31
Punjab		30,141,373	-11,415	36,247	-37.873	120.255	6/30
Rajasthan		81,032,689	6,629	32,311	8.181	39.874	5/31
Tamil Nadu	Chennai	7,088,000	5,921	19,199	83.540	270.866	8/01
	Entire state	77,841,270	72,052	206,274	92.563	264.993	8/31
	6 cities	5,980,370	-1,032	14,160	-17.261	236.776	5/31
	Madurai	3,038,250	2,018	2,325	66.412	76.508	5/31
Telangana	Hyderabad	9,482,000	8,359	6,805	88.157	71.768	5/31
Uttar Pradesh		237,882,725	-37,468	48,458	-15.751	20.371	4/30
West Bengal	Entire state	99,609,300	53,355	49,122	53.564	49.315	5/31
	Kolkata	4,496,694	2,075	2,901	46.145	64.514	5/23

Data sources listed in supplemental references for: Andhra Pradesh (Banaji), Delhi (Radhakrishnan), Kolkata (Karlinsky), Bengaluru (Chatterjee 2021), Tamil Nadu (crstn.org), Madurai district (Radhakrishnan 2021), Kerala (devdatalab), Uttar Pradesh data (Das), Rajasthan (devdatalab), Haryana (Ramani), Punjab (Ramani), and Himachal Pradesh (Ramani). Gujarat 2020 data available Jan.-Nov. Ahmedabad 2020 data available Apr.-May. Gujarat, including Ahmedabad, 2021 mortality available from Mar 1 to May 10, 2021. Death register data from 68 municipalities in Gujarat, with 6.01% of state population, available through April 2021 (Jalihal 2021). Mortality data for May 1 to June 10, 2021 were available from 3 municipalities in Gujarat (Chorvad, Idar, Khedbrahma) with 0.131% of the state population (wallofgrief.org). Nagpur data available Apr. to Dec. 2020. The 6 government hospitals in Tamil Nadu studied by Arappor Iyakkam from Jan-May in 2020 and 2021 were in Madurai, Coimbatore, Trichy, Vellore, Karur, and Tirupur. The urban areas in Madhya Pradesh from which funeral counts were tabulated for April 2021 were from the following 25 districts: Barwani, Bhind, Bhopal, Burhanpur, Chhatarpur, Chhindwara, Dewas, Dhar, Gwalior, Indore, Jabalpur, Jhabua, Khandwa, Mandsaur, Morena, Neemuch, Ratlam, Sagar, Satna, Seoni, Shahdol, Shivpuri, Singroli, Tikamgarh, Vidisha (Datta 2021).

Maharashtra data did not include 6 districts: Pune, Satara, Sangli, Yavatmal, Sindhudurg and Gondiya (Ramani). State populations were obtained from the Johns Hopkins University Center for Systems Science and Engineering dataset.

Supplemental Table S2. Time Series of Mortality from Selected Regions in India.

Region	Year	Period	Deaths	Regist-ration Comp-leteness	Reference
Andhra Pradesh	2015	All	310,640	0.849	Vital Statistics
Andhra Pradesh	2016	All	313,285	0.887	Vital Statistics
Andhra Pradesh	2017	All	355,546	0.944	Vital Statistics
Andhra Pradesh	2018	All	375,777	1.000	Vital Statistics
Andhra Pradesh	2019	All	401,472	1.000	Vital Statistics
Andhra Pradesh	2018	All	333,275		Rukmini "AP"
Andhra Pradesh	2019	All	363,414		Rukmini "AP"
Andhra Pradesh	2020	All	428,907		Rukmini "AP"
Andhra Pradesh	2018	Months 1-7	193,698		Banaji (github)
Andhra Pradesh	2019	Months 1-7	206,461		
Andhra Pradesh	2020	Months 1-7	198,030		
Andhra Pradesh	2021	Months 1-7	385,608		
Assam State	2015	All	116,778	0.511	Vital Statistics
Assam State	2016	All	130,414	0.598	Vital Statistics
Assam State	2017	All	141,012	0.659	Vital Statistics
Assam State	2018	All	142,605	0.669	Saikia, Vital St.
Assam State	2019	All	163,057	0.740	Saikia, Vital St.
Assam State	2020	All	187,085		Saikia
Bihar	2015	All	204,093	0.319	Vital Statist.
Bihar	2016	All	177,021	0.283	Vital Statist.
Bihar	2017	All	261,425	0.427	Vital Statist.
Bihar	2018	All	213,989	0.346	Vital Statist.
Bihar	2019	All	359,349	0.516	Vital Statist.
Bihar	2018	Entire year	182,921		devdatalab
	2019	Entire year	351,274		devdatalab
	2020	Entire year	387,429		devdatalab
Bihar	2018	Jan-May	45,487		devdatalab
	2019	Jan-May	133,224		devdatalab

	2020	Jan-May	138,693		devdatalab
	2021	Jan-May	215,746		devdatalab
Chandigarh	2015	Entire year	16,203	1.000	Vital Statistics
Chandigarh	2016	Entire year	16,570	1.000	Vital Statistics
Chandigarh	2017	Entire year	21,236	1.000	Vital Statistics
Chandigarh	2018	Entire year	23,330	1.000	Vital Statistics
Chandigarh	2019	Entire year	23,592	1.000	Vital Statistics
Chandigarh	2018	Entire year	23,228		Banaji
Chandigarh	2019	Entire year	23,352		Banaji
Chandigarh	2020	Entire year	18,215		Banaji
Chandigarh	2018	Jan-May	9,367		Banaji
Chandigarh	2019	Jan-May	9,748		Banaji
Chandigarh	2020	Jan-May	7,641		Banaji
Chandigarh	2021	Jan-May	9,026		Banaji
Chhattisgarh	2018	Entire year	15,690		Online portal of the state CRS (devdatalab)
Chhattisgarh	2019	Entire year	15,607		
Chhattisgarh	2020	Entire year	63,237		
Chhattisgarh	2018	Months 1-5	6,802		Online portal of the state CRS (devdatalab)
Chhattisgarh	2019	Months 1-5	5,623		
Chhattisgarh	2020	Months 1-5	17,497		
Chhattisgarh	2021	Months 1-5	106,832		
Chhattisgarh	2015	Entire year	168,034	0.875	Vital Statistics
Chhattisgarh	2016	Entire year	182,985	0.952	Vital Statistics
Chhattisgarh	2017	Entire year	175,035	0.888	Vital Statistics
Chhattisgarh	2018	Entire year	177,549	0.835	Vital Statistics
Chhattisgarh	2019	Entire year	188,211	0.815	Vital Statistics
Delhi	2015	All	124,516	1.000	Vital Statistics
Delhi	2016	All	141,632	1.000	Vital Statistics
Delhi	2017	All	136,117	1.000	Vital Statistics
Delhi	2018	All	145,533	1.000	Vital Statistics
Delhi	2019	All	145,284	1.000	Vital Statistics
Delhi	2019	Apr-May	19,047		Hindust. Tim.
Delhi	2020	Apr-May	10,258		Hindust. Tim.
Delhi	2021	Apr-May	33,109		Hindust. Tim.
Delhi	2018	Entire year	145,533		Radhakrishnan
Delhi	2019	Entire year	145,284		Radhakrishnan
Delhi	2020	Entire year	153,449		Radhakrishnan
Delhi	2018	Jan-Jun	74,063		Radhakrishnan
Delhi	2019	Jan-Jun	74,464		Radhakrishnan
Delhi	2020	Jan-Jun	70,144		Radhakrishnan
Delhi	2021	Jan-Jun	123,176		Radhakrishnan
Gujarat	2015	All	412,322	1.000	Vital Statistics
Gujarat	2016	All	417,835	1.000	Vital Statistics
Gujarat	2017	All	388,316	0.982	Vital Statistics
Gujarat	2018	All	433,256	1.000	Vital Statistics

Gujarat	2019	All	462,284	1.000	Vital Statistics
Gujarat	2017	Jan 1-Nov 30	368,000		Dave
Gujarat	2018	Jan 1-Nov 30	393,000		Dave
Gujarat	2019	Jan 1-Nov 30	419,000		Dave
Gujarat	2020	Jan 1-Nov 30	374,000		Dave
Gujarat	2020	Mar. 1-May 10	58,000		Desai
Gujarat	2021	Mar. 1-May 10	123,871		Desai
Gujarat: 68 municipalities	2019	Entire year	33,362		Jalihal
	2020	Entire year	40,117		Jalihal
	2019	Jan-Apr	11,354		Jalihal
	2020	Jan-Apr	11,364		Jalihal
	2021	Jan-Apr	21,460		Jalihal
Gujarat: Chorvad, Idar, Khedbrahma	2019	May 1-June 10	82		wallofgrief.org
	2020		96		wallofgrief.org
	2021		228		wallofgrief.org
Gujarat: Ahmedabad	2017	Mar. 1-May 10	9,319		Opindia
	2018	Mar. 1-May 10	9,866		Opindia
	2019	Mar. 1-May 10	9,950		Opindia
	2020	Mar. 1-May 10	7,786		Opindia
	2021	Mar. 1-May 10	13,593		Opindia
Gujarat: Ahmedabad	2019	Apr-May	5,490		Khanna
	2020	Apr-May	10,708		Khanna
Haryana	2015	Entire year	168,910	1.000	Vital Statistics
	2016	Entire year	181,138	1.000	Vital Statistics
	2017	Entire year	174,937	1.000	Vital Statistics
	2018	Entire year	185,842	1.000	Vital Statistics
	2019	Entire year	188,910	1.000	Vital Statistics
Haryana	2018	Entire year	178,536		Ramani
	2019	Entire year	184,155		Ramani
	2020	Entire year	198,223		Ramani
Haryana	2018	Jan-May	74,816		Ramani
	2019	Jan-May	76,870		Ramani
	2020	Jan-May	78,030		Ramani
	2021	Jan-May	121,100		Ramani

Himachal Pradesh	2015	Entire year	41,462	0.882	Vital statistics
	2016	Entire year	35,819	0.734	Vital statistics
	2017	Entire year	39,114	0.821	Vital statistics
	2018	Entire year	41,833	0.834	Vital statistics
	2019	Entire year	43,633	0.864	Vital statistics
Himachal Pradesh	2018	Entire year	42,151		Ramani
	2019	Entire year	42,989		Ramani
	2020	Entire year	44,436		Ramani
Himachal Pradesh	2018	Jan-May	17,759		Ramani
	2019	Jan-May	17,515		Ramani
	2020	Jan-May	16,806		Ramani
	2021	Jan-May	21,180		Ramani
Karnataka: Bengaluru	2019	Entire year	65,019		devdatalab
	2020	Entire year	75,441		devdatalab
Karnataka: Bengaluru	2019	Jan-June	31,726		devdatalab
	2020	Jan-June	28,830		devdatalab
	2021	Jan 1-June 15	87,082		Chatterjee
Karnataka	2015	All	393,731	0.962	Srivat., Vital Statistics, devdatalab (all agree)
Karnataka	2016	All	420,774	1.000	
Karnataka	2017	All	481,747	1.000	
Karnataka	2018	All	483,511	1.000	
Karnataka	2019	All	508,584	1.000	
Karnataka	2020	All	551,808		Srivatsa, devdatalab
Karnataka	2018	Jan-June 15	224,000		Chatterjee
Karnataka	2019	Jan-June 15	235,000		Chatterjee
Karnataka	2021	Jan-June 15	337,580		Chatterjee
Karnataka	2015	Jan-Aug	260,513		devdatalab
Karnataka	2016	Jan-Aug	278,351		devdatalab
Karnataka	2017	Jan-Aug	309,995		devdatalab
Karnataka	2018	Jan-Aug	323,028		devdatalab
Karnataka	2019	Jan-Aug	324,531		devdatalab
Karnataka	2020	Jan-Aug	327,917		devdatalab
Karnataka	2021	Jan-Aug	474,070		karnataka.gov
Kerala	2015	Jan 1-May 31	95,281		devdatalab
Kerala	2016	Jan 1-May 31	98,195		devdatalab
Kerala	2017	Jan 1-May 31	96,534		devdatalab
Kerala	2018	Jan 1-May 31	99,473		devdatalab

Kerala	2019	Jan 1-May 31	104,705		devdatalab
Kerala	2020	Jan 1-May 31	95,544		devdatalab
Kerala	2021	Jan 1-May 31	113,372		devdatalab
Kerala	2015	Entire year	235,982		Rajendran
Kerala	2016	Entire year	244,900		Rajendran
Kerala	2017	Entire year	252,103		Rajendran
Kerala	2018	Entire year	255,594		Rajendran
Kerala	2019	Entire year	264,150		Rajendran
Kerala	2020	Entire year	252,421		Rajendran
Kerala	2021	Jan 1-May 31	113,372		Rajendran
Kerala	2015	All	252,576	1.000	Vital Statistics
Kerala	2016	All	256,130	0.943	Vital Statistics
Kerala	2017	All	263,342	1.000	Vital Statistics
Kerala	2018	All	258,530	1.000	Vital Statistics
Kerala	2019	All	270,567	1.000	Vital Statistics
Kerala	2015	All	236,859		lsgkerala.gov
Kerala	2016	All	244,894		lsgkerala.gov
Kerala	2017	All	252,097		lsgkerala.gov
Kerala	2018	All	255,571		lsgkerala.gov
Kerala	2019	All	264,131		lsgkerala.gov
Kerala	2020	All	242,910		lsgkerala.gov
Kerala	2021	Jan 1-Aug 31	166,514		lsgkerala.gov
Madhya Pradesh	2015	All	311,411	0.538	Vital Statistics
Madhya Pradesh	2016	All	338,587	0.609	Vital Statistics
Madhya Pradesh	2017	All	370,538	0.687	Vital Statistics
Madhya Pradesh	2018	All	424,527	0.788	Vital Statistics
Madhya Pradesh	2019	All	493,328	0.891	Vital Statistics
Madhya Pradesh	2018	Months 1-12	407,172		LM
Madhya Pradesh	2019	Months 1-12	449,819		LM
Madhya Pradesh	2020	Months 1-12	461,057		LM
Madhya Pradesh	2018	Months 1-5	152,346		LM

Madhya Pradesh	2019	Months 1-5	167,549		LM
Madhya Pradesh	2020	Months 1-5	164,191		LM
Madhya Pradesh	2021	Months 1-5	348,708		LM
Madhya Pradesh: urban regions of 25 districts	2015	Entire year	119,068		Vital Statistics
	2016	Entire year	112,303		Vital Statistics
	2017	Entire year	113,612		Vital Statistics
	2018	Entire year	112,188		Vital Statistics
	2019	Entire year	119,914		Vital Statistics
	2021	April 1-30	21,456		Datta
Maharashtra	2015	Entire year	673,824	0.975	Vital Statistics
Maharashtra	2016	Entire year	666,448	0.937	Vital Statistics
Maharashtra	2017	Entire year	647,161	0.931	Vital Statistics
Maharashtra	2018	Entire year	667,900	0.984	Vital Statistics
Maharashtra	2019	Entire year	693,800	1.000	Vital Statistics
Maharashtra (less 6 districts)	2018	Entire year	426,489		Ramani
	2019	Entire year	462,028		Ramani
	2020	Entire year	565,180		Ramani
	2018	Jan-May	177,130		Ramani
	2019	Jan-May	186,004		Ramani
	2020	Jan-May	197,676		Ramani
	2021	Jan-May	287,123		Ramani
Maharashtra: Mumbai City	2017	Entire year	89,037		WM
	2018	Entire year	88,852		WM
	2019	Entire year	91,123		WM
	2020	Entire year	109,398		WM
Maharashtra: Mumbai City	2017	Jan-May	36,166		WM
	2018	Jan-May	37,173		WM
	2019	Jan-May	37,363		WM
	2020	Jan-May	35,914		WM
	2021	Jan-May	45,163		WM
Maharashtra: Nagpur City	2019	Months 4-12	16,238		WM
	2020	Months 4-12	20,382		WM
Odisha	2015	All year	321,009	1.000	Vital statistics
Odisha	2016	All year	345,527	1.000	Vital statistics
Odisha	2017	All year	322,660	1.000	Vital statistics
Odisha	2018	All year	328,799	1.000	Vital statistics
Odisha	2019	All year	342,947	1.000	Vital statistics
Odisha	2020	All year	362,982		Mohanty
Odisha	2021	Jan-Aug	286,623		odisha.gov.in
Punjab	2015	Entire year	199,461	1.000	Vital statistics

Punjab	2016	Entire year	213,578	1.000	Vital statistics
Punjab	2017	Entire year	210,398	1.000	Vital statistics
Punjab	2018	Entire year	213,234	1.000	Vital statistics
Punjab	2019	Entire year	215,045	1.000	Vital statistics
Punjab	2015	Entire year	199,749		Ramani
Punjab	2016	Entire year	210,848		Ramani
Punjab	2017	Entire year	208,005		Ramani
Punjab	2018	Entire year	211,213		Ramani
Punjab	2019	Entire year	213,122		Ramani
Punjab	2020	Entire year	228,136		Ramani
Punjab	2015	Jan-June	100,258		Ramani
Punjab	2016	Jan-June	101,885		Ramani
Punjab	2017	Jan-June	101,688		Ramani
Punjab	2018	Jan-June	105,856		Ramani
Punjab	2019	Jan-June	107,823		Ramani
Punjab	2020	Jan-June	106,371		Ramani
Punjab	2021	Jan-June	147,389		Ramani
Rajasthan	2015	Entire year	409,463	0.899	Vital statistics
Rajasthan	2016	Entire year	416,992	0.933	Vital statistics
Rajasthan	2017	Entire year	424,763	0.953	Vital statistics
Rajasthan	2018	Entire year	443,173	0.999	Vital statistics
Rajasthan	2019	Entire year	451,315	0.986	Vital statistics
Rajasthan	2018	Entire year	216,370		Ramani
Rajasthan	2019	Entire year	219,814		Ramani
Rajasthan	2020	Entire year	229,564		Ramani
Rajasthan	2018	Months 1-5	95,484		Ramani
Rajasthan	2019	Months 1-5	90,366		Ramani
Rajasthan	2020	Months 1-5	94,566		Ramani
Rajasthan	2021	Months 1-5	125,863		Ramani
Tamil Nadu, 6 cities: Madurai, Coimbatore, Trichy, Vellore, Karur, Tirupur.	2019	Jan-May	10,587		Nagar
	2020	Jan-May	9,432		Nagar
	2021	Jan-May	18,587		Nagar
Tamil Nadu: Madurai District.	2019	All	19,735		Radhakrishnan
	2020	All	21,524		Radhakrishnan
	2019	Jan-May	8,789		Radhakrishnan
	2020	Jan-May	7,724		Radhakrishnan
	2021	Jan-May	11,208		Radhakrishnan
Tamil Nadu	2015	All year	568,271	1.000	Vital Statistics
	2016	All year	563,625	1.000	Vital Statistics
	2017	All year	580,496	1.000	Vital Statistics
	2018	All year	574,006	1.000	Vital Statistics
	2019	All year	633,897	1.000	Vital Statistics
Tamil Nadu	2018	Entire year	549,209		crstn.org
	2019	Entire year	637,270		crstn.org

	2020	Entire year	687,488		crstn.org
	2021	Jan-Aug	607,307		crstn.org
Tamil Nadu	2018	All year	536,192		LM; Ramani
	2019	All year	588,221		LM; Ramani
	2020	All year	644,291		LM; Ramani
	2018	Jan-Aug	363,744		LM; Ramani
	2019	Jan-Aug	392,304		LM; Ramani
	2020	Jan-Aug	426,100		LM; Ramani
Tamil Nadu: Chennai	2015	Wks 1-52	59,875		LM
	2016	Wks 1-52	57,826		LM
	2017	Wks 1-52	63,726		LM
	2018	Wks 1-52	62,793		LM
	2019	Wks 1-52	67,002		LM
	2020	Wks 1-52	73,932		LM
	2015	Wks 1-30	33,691		Rukmini
	2016	Wks 1-30	33,837		Rukmini
	2017	Wks 1-30	36,056		Rukmini
	2018	Wks 1-30	35,927		Rukmini
	2019	Wks 1-30	38,145		Rukmini
	2020	Wks 1-30	40,338		Rukmini
	2021	Wks 1-30	59,129		Rukmini
Telangana	2015		192,857	0.819	Vital Statistics
	2016		204,917	0.920	Vital Statistics
	2017		178,345	0.735	Vital Statistics
	2018		136,528	0.585	Vital Statistics
	2019		228,294	0.972	Vital Statistics
Telangana: Hyderabad	2016	Jan-Dec	49,523		LM, Ramani
	2017	Jan-Dec	52,710		LM, Ramani
	2018	Jan-Dec	55,026		LM, Ramani
	2019	Jan-Dec	66,131		LM, Ramani
	2020	Jan-Dec	77,241		LM, Ramani
Telangana: Hyderabad	2016	Jan-May	18,839		LM, Ramani
	2017	Jan-May	20,645		LM, Ramani
	2018	Jan-May	21,696		LM, Ramani
	2019	Jan-May	25,657		LM, Ramani
	2020	Jan-May	24,884		LM, Ramani
	2021	Jan-May	36,041		LM, Ramani
Uttar Pradesh	2015	Entire year	687,416	0.442	Vital Statistics
Uttar Pradesh	2016	Entire year	608,740	0.402	Vital Statistics
Uttar Pradesh	2017	Entire year	571,170	0.383	Vital Statistics
Uttar Pradesh	2018	Entire year	906,653	0.608	Vital Statistics
Uttar Pradesh	2019	Entire year	944,596	0.633	Vital Statistics
Uttar Pradesh	2019	Entire year	773,402		Bhawan
Uttar Pradesh	2020	Entire year	793,505		Bhawan
Uttar Pradesh	2019	Jan 1-Apr	259,316		Bhawan

		30			
Uttar Pradesh	2020	Jan 1-Apr 30	188,747		Bhawan
Uttar Pradesh	2021	Jan 1-Apr 30	333,878		Bhawan
West Bengal	2015	Entire year	403,180	0.723	Vital statistics
West Bengal	2016	Entire year	445,540	0.806	Vital statistics
West Bengal	2017	Entire year	442,995	0.797	Vital statistics
West Bengal	2018	Entire year	490,530	0.908	Vital statistics
West Bengal	2019	Entire year	551,695	1.000	Vital statistics
West Bengal	2018	Entire year	368,477		Ramani
West Bengal	2019	Entire year	410,503		Ramani
West Bengal	2020	Entire year	463,858		Ramani
West Bengal	2018	Jan-May	155,108		Ramani
West Bengal	2019	Jan-May	167,422		Ramani
West Bengal	2020	Jan-May	176,451		Ramani
West Bengal	2021	Jan-May	216,972		Ramani
West Bengal: Kolkata	2015	Weeks 1-52	62,710		LM
	2016	Weeks 1-52	65,060		LM
	2017	Weeks 1-52	69,910		LM
	2018	Weeks 1-52	68,998		LM
	2019	Weeks 1-52	69,844		LM
	2020	Weeks 1-52	74,841		LM
West Bengal: Kolkata	2015	Weeks 1-20	25,128		LM
	2016	Weeks 1-20	24,506		LM
	2017	Weeks 1-20	26,980		LM
	2018	Weeks 1-20	25,985		LM
	2019	Weeks 1-20	29,222		LM
	2020	Weeks 1-20	27,674		LM
	2021	Weeks 1-20	33,132		LM

LM = Local Mortality dataset (Karlinsky 2021).

The urban areas in Madhya Pradesh from which funeral counts were tabulated for April 2021 were from the following 25 districts: Barwani, Bhind, Bhopal, Burhanpur, Chhatarpur, Chhindwara, Dewas, Dhar, Gwalior, Indore, Jabalpur, Jhabua, Khandwa, Mandsaur, Morena, Neemuch, Ratlam, Sagar, Satna, Seoni, Shahdol, Shivpuri, Singroli, Tikamgarh, Vidisha (Datta 2021).

Supplemental Table S3. Covid-19 Deaths Based on Viral Testing and Clinical Symptoms, as Tabulated by Johns Hopkins University.

Region.	Population	Reported Covid Deaths in 2020	Deaths/ 100K in 2020	Reported Covid deaths by August 31, 2021.	Deaths/ 100K in 2021 (as of August 31)
Andhra Pradesh	53,903,393	7,104	13.179	13,838	12.493
Assam	35,607,039	1,043	2.929	5,655	12.952
Bihar	124,799,930	1,393	1.116	9,653	6.619
Chandigarh	11,584,730	316	2.728	813	4.290
Delhi	18,710,920	10,523	56.240	25,081	77.805
Gujarat	63,872,400	4,302	6.735	10,081	9.048
Haryana	28,204,692	2,899	10.278	9,675	24.024
Himachal Pradesh	7,451,955	931	12.493	3,595	35.749
Karnataka	67,562,700	12,081	17.881	37,293	37.316
Kerala	35,699,440	3,042	8.521	20,673	49.387
Madhya Pradesh	85,358,970	3,595	4.212	10,516	8.108
Maharashtra	123,144,200	49,463	40.167	137,209	71.255
Odisha	46,356,334	1,871	4.036	7,901	13.008
Punjab	30,141,373	5,331	17.687	16,373	36.634
Rajasthan	81,032,689	2,689	3.318	8,954	7.731
Tamil Nadu	77,841,270	12,109	15.556	34,899	29.278
Telangana	39,362,732	1,541	3.915	3,872	5.922
Uttar Pradesh	237,882,725	8,352	3.511	22,820	6.082
West Bengal	99,609,300	9,683	9.721	18,434	8.785
Total, except Uttar Pradesh	1,030,244,067	129,916	12.610	374,515	23.742
Total, except Utt. Prad. & Assam	994,637,028	--	--	368,860	24.128
Total, except Assam	1,232,519,753	--	--	391,680	20.645
Total	1,268,126,792	138,268	10.903	397,335	20.429

This table lists absolute and per-capita reported Covid-19 deaths, rather than excess deaths.

Supplemental Table S4. Range of Estimated Covid-19 Deaths in India, 2020 through August 2021.

State	Population	Estimate Type.	2020		2021, by August 31	
			Deaths/ 100K	Deaths	Deaths/ 100K	Deaths
Andhra Prad.	53,903,393	--	121.501	65,493	321.772	173,446
Assam	35,607,039	--	42.819	15,246	--	--
Bihar	124,799,930	--	107.567	134,244	101.272	126,388
Chandigarh	11,584,730	--	2.728	316	4.290	813
Delhi	18,710,920	--	56.240	10,523	230.342	43,099
Gujarat	63,872,400	Low	6.735	4,302	36.417	23,260
		High	182.464	116,544	430.389	274,900
Haryana	28,204,692	--	39.311	11,087	145.458	41,026
Himachal Pradesh	7,451,955	--	12.493	931	39.930	2,976
Karnataka	67,562,700	Low	18.689	12,627	165.588	111,876
		High	77.103	52,093	648.291	438,003
Kerala	35,699,440	--	8.521	3,042	49.387	17,631
Madhya Pradesh	85,358,970	Low	4.212	3,595	46.376	39,586
		High	4.212	3,595	212.232	181,159
Maharashtra	123,144,200	Low	100.540	103,152	71.255	137,209
		High	172.308	212,187	102.288	104,946
Odisha	46,356,334	--	43.220	20,035	125.631	58,238
Punjab	30,141,373	--	17.687	5,331	120.255	36,247
Rajasthan	81,032,689	--	8.181	6,629	39.874	32,311
Tamil Nadu	77,841,270	Low	15.556	12,109	76.508	59,555
		High	92.563	72,052	270.866	210,846
Telangana	39,362,732	--	88.157	34,701	71.768	28,250
Uttar Prad.	237,882,725	--	3.511	8,352	20.371	48,458
West Bengal	99,609,300	Low	46.145	45,965	49.315	49,122
		High	53.564	53,355	64.514	64,262
Total, except Uttar Prad.	1,030,244,067	Low	47.496	489,328	--	--
		High	79.341	817,404	--	--
Total, except Uttar Prad.	994,637,028	Low	--	--	98.632	981,033
		High	--	--	184.443	1,834,540
Total	1,268,126,792	Low	39.245	497,680	--	--
		High	65.116	825,756	--	--
	1,232,519,753	Low	--	--	83.527	1,029,491
		High	--	--	152.776	1,882,998

All estimates based on excess deaths, except 2020 Delhi, Kerala, and Punjab data, which are based on known Covid-19 mortality as reported by Johns Hopkins University, and Gujarat, West Bengal, Madhya Pradesh, and Tamil Nadu, which included the Hopkins data as the low end of the uncertainty range.

Supplemental References.

Alavi AM. NDTV. Bihar Saw Nearly 75,000 Unaccounted Deaths Amid 2nd Covid Wave, Data Shows. June 19, 2021. Available from: https://www.ndtv.com/india-news/bihar-saw-nearly-75-000-unaccounted-deaths-amid-2nd-covid-wave-data-shows-2467778?pfrom=home-ndtv_topscroll Accessed June 20, 2021.

Anparthi A. 128 more deaths than normal ones, Covid fatalities in Dec '20 [in Nagpur]. Times of India. January 21, 2021. Available from: https://timesofindia.indiatimes.com/city/nagpur/128-more-deaths-than-normal-ones-covid-fatalities-in-dec-20/articleshow/80373223.cms Accessed June 16, 2021.

Banaji M. Indian All-Cause Mortality Data. Available from: https://github.com/muradbanaji/IndiaACMdata Accessed September 8, 2021.

Bhawan S. Deputy Chief Registrar. Vital Dept (Birth & Death). Government of Uttar Pradesh. Information furbished for RTI [requested by Saurav Das]. June 2, 2021.

Chatterjee S. Karnataka recorded 1.02 lakh 'excess' deaths in 2021, 5 times the COVID-19 toll. The News Minute. June 15, 2021. Available from: https://www.thenewsminute.com/article/karnataka-recorded-102-lakh-excess-deaths-2021-5-times-covid-19-toll-150761 Accessed June 16, 2021.

Das S. Death Count In 24 UP Districts 43 Times More Than Official Covid-19 Toll. Article14. June 21, 2021. Available from: https://article-14.com/post/untitled-60cf605395758 Accessed June 21, 2021.

Datta S. Madhya Pradesh Excess Deaths in April: An Alternative Count Using Funeral Data. Thewire.in. June 22, 2021. Available from: https://science.thewire.in/health/madhya-pradesh-excess-deaths-covid-19-april-2021-funeral-data/ Accessed June 26, 2021.

Dave K. Year of pandemic? Gujarat records 11% less deaths than in 2019. Times of India. December 13, 2020. Available from: https://timesofindia.indiatimes.com/city/ahmedabad/year-of-pandemic-gujarat-records-11-less-deaths-than-in-2019/articleshow/79704719.cms Accessed June 19, 2021.

Debroy S. 13,000 more deaths in Mumbai this year between March & September. Times of India. October 29, 2020. Available from: https://timesofindia.indiatimes.com/city/mumbai/13k-more-deaths-in-city-this-year-between-march-sept/articleshow/78920631.cms Accessed June 27, 2021.

Desai D. 61k Covid deaths not counted in Gujarat: Report. Hindustan Times. May 15, 2021. Available from: https://www.hindustantimes.com/india-news/61k-covid-deaths-not-counted-in-gujarat-report-101621027267608.html Accessed June 14, 2021.

Government of India. Office of the Registrar General. Ministry of Home Affaris. Vital Statistics of India. Based on the Civil Registration System. 2019. June 2021. Available from: https://crsorgi.gov.in/web/uploads/download/CRS%202019%20report.pdf

Accessed June 18, 2021.

Government of India. Office of the Registrar General. Ministry of Home Affaris. Vital Statistics of India. Based on the Civil Registration System. 2018. June 2020.

https://censusindia.gov.in/2011-Common/CRS_2018/crs2018_20072020.pdf

Accessed June 18, 2021.

Government of India. Office of the Registrar General. Ministry of Home Affaris. Vital Statistics of India. Based on the Civil Registration System. 2015. June 2017. Available from: https://censusindia.gov.in/2011-Documents/CRS_Report/crs_report%202015_23062017.pdf Accessed June 18, 2021.

Government of India. Office of the Registrar General. Ministry of Home Affaris. Vital Statistics of India. Based on the Civil Registration System. 2016. June 2018. Available from: https://censusindia.gov.in/2011-Documents/CRS_Report/CRS%20FINAL%20REPORT%202016_21062018.pdf Accessed June 18, 2021.

Government of Karnataka. Annual Report on the Registration of Births and Deaths Act, 1969. [2015-2019]. Available from: https://ejanma.karnataka.gov.in/frmVitalSat.aspx

Accessed September 1, 2021.

Government of Karnataka. Office of the Chief Registrar of Births and Deaths. Registration Details. Available from: https://ejanma.karnataka.gov.in/frmTransaction_Details.aspx Accessed September 1, 2021.

Government of Kerala. Civil Registrations. June 20, 2021. Available from: https://cr.lsgkerala.gov.in/Pages/map.php Accessed September 1, 2021.

Government of Odisha. Health and Family Welfare Department. Birth and Death Odisha. Available from: https://www.birthdeath.odisha.gov.in/#/home Accessed September 1, 2021.

Government of Tamil Nadu. Birth and Death Registration. June 27, 2021 [in India]. Available from: http://www.crstn.org/birth_death_tn/MisRep.jsp Accessed September 1, 2021.

[The Hindu] Special Correspondent. 32% increase in mortality rate in Bengaluru: Patil. The Hindu. Sep. 3, 2020. Available from: https://www.thehindu.com/news/national/karnataka/32-increase-in-mortality-rate-in-bengaluru-patil/article32509298.ece Accessed: June 16, 2021.

Hindustan Times Correspondent. What do Delhi’s death registration figures tell us about Covid-19 death toll? Hindustan Times. June 4, 2021. Available from: https://www.msn.com/en-in/news/other/what-do-delhi-s-death-registration-figures-tell-us-about-covid-19-death-toll/ar-AAKHLUM Accessed June 6, 2021.

IANS. COVID-19 year 2021 sees 1.4L more deaths than 2019 in Tamil Nadu, says a study by NGO [Arappor Iyakkam]. The Free Press Journal. June 26, 2021. Available from: https://www.freepressjournal.in/india/covid-19-year-2021-sees-14l-more-deaths-than-2019-in-tamil-nadu-says-a-study-by-ngo Accessed June 26, 2021.

Jalihal S, Sethi T, Sethi N. Gujarat: Data from death registers suggests COVID toll undercounted by 27 times. The Wire. August 17, 2021. Available from: https://thewire.in/health/gujarat-data-death-registers-covid-19-toll-undercounted-27-times Accessed September 11, 2021.

Khanna S. Other deaths spike in Indian city [Ahmedabad] ravaged by coronavirus. Reuters. July 2, 2020. Available from: https://www.reuters.com/article/us-health-coronavirus-india-casualties-idUSKBN24311A Accessed June 16, 2021.

Menon JC, Rakesh PS, Bharti OK, Mishra K, Swain BK, Raina SK, John D, Janakiraman C, Banerjee A. COVID-19 and all-cause-mortality; differential trends between countries and selected states of India. April 15, 2021. Available from: https://www.researchsquare.com/article/rs-361210/v1 Accessed September 6, 2021.

Mohanty D. Odisha: 20,035 excess deaths in 2020 amid Covid deaths underreporting cloud. Hindustan Times. September 12, 2021. Available from: https://www.hindustantimes.com/india-news/odisha-20-035-excess-deaths-in-2020-amid-covid-deaths-underreporting-cloud-101631417118338.html Accessed September 12, 2021.

Nagar T. Citizen’s Research Report on Covid Deaths in TamilNadu. Report by Arappor Iyakkam. June 15, 2021. Available from: https://arappor.org/public/Document/20210615_Citizensreport_Coronadeath_withAnnexure.pdf Accessed June 16, 2021.

OpIndia Staff. Gujarat: Here’s why the death certificate stats from March to May so far do not prove allegations of massive undercounting of Covid deaths. OpIndia. May 15, 2021. Available from: https://www.opindia.com/2021/05/gujarat-covid-19-deaths-death-certificate-statistics-divya-bhaskar/ Accessed June 14, 2021. [Has Gujarat and Ahmedabad data.]

Radhakrishnan S. Is Madurai undercounting Covid deaths? Data raise questions. The New Indian Express. June 18, 2021. Available from: https://www.newindianexpress.com/states/tamil-nadu/2021/jun/18/is-madurai-undercounting-covid-deaths-data-raise-questions-2317785.html Accessed June 17, 2021.

Radhakrishnan V. ‘Excess deaths’ in Delhi two times official COVID-19 toll. The Hindu. September 3, 2021. Available from: https://www.thehindu.com/news/cities/Delhi/excess-deaths-in-delhi-two-times-official-covid-19-toll/article36279423.ece Accessed September 8, 2021.

Rajendran D, Kurian S. TNM Exclusive: Kerala reports around 14,000 excess deaths till end of May 2021. The News Minute. Available from: https://www.thenewsminute.com/article/tnm-exclusive-kerala-reports-almost-14000-excess-deaths-till-end-may-2021-151124 Accessed June 23, 2022.

Ramani S, Radhakrishnan V. Excess deaths in Hyderabad are 10 times the official COVID-19 toll for Telangana. The Hindu. June 14, 2021. Available from: https://www.thehindu.com/news/cities/Hyderabad/excess-deaths-in-hyderabad-are-10-times-the-official-covid-19-toll-for-telangana/article34807214.ece Accessed June 14, 2021.

Ramani S, Radhakrishnan V. Number of death certificates issued by GHMC in a month. Available from: https://docs.google.com/spreadsheets/d/1wP1SOEHclYOmA2hokjBzjCAO_eqpvx9LPFtxDOkv1zg/edit#gid=0 Accessed June 14, 2021.

Ramani S, Kannan R. Excess deaths in Tamil Nadu over four times official COVID-19 tally. The Hindu. June 16, 2021. https://www.thehindu.com/news/national/tamil-nadu/excess-deaths-in-tamil-nadu-over-four-times-official-covid-19-tally/article34834150.ece?homepage=true Accessed June 17, 2021.

Ramani S. Excess deaths in Rajasthan are at least five times the official COVID-19 tally. The Hindu. July 4, 2021. Available from: https://www.thehindu.com/news/national/excess-deaths-in-rajasthan-are-five-times-the-official-covid-19-tally/article35118826.ece Accessed July 23, 2021.

Ramani S. ‘Excess deaths’ in Haryana seven times official COVID-19 toll. The Hindu. July 14, 2021. Available from: https://www.thehindu.com/news/national/excess-deaths-in-haryana-seven-times-official-covid-19-toll/article35329023.ece Accessed July 24, 2021.

Ramani S, Vasudeva V. Punjab ‘excess deaths’ three times official toll. The Hindu. July 30, 2021. Available from: https://www.thehindu.com/news/national/coronavirus-punjab-excess-deaths-three-times-official-toll/article35641394.ece?homepage=true Accessed July 31, 2021.

Ramani S. Himachal Pradesh ‘excess deaths’ twice the official COVID-19 toll. The Hindu. July 21, 2021. Available from: https://www.thehindu.com/news/national/himachal-pradesh-excess-deaths-twice-the-official-covid-19-toll/article35430252.ece Accessed July 31, 2021.

Ramani S. Excess deaths in West Bengal 11 times official COVID-19 tally. The Hindu. July 25, 2021. Available from: https://www.thehindu.com/news/national/other-states/excess-deaths-in-west-bengal-11-times-official-covid-19-tally/article35526895.ece Accessed July 31, 2021.

Ramani S. Excess deaths in Maharashtra were at least 3 times the official COVID toll. The Hindu. August 3, 2021. Available from: https://www.thehindu.com/news/national/excess-deaths-in-maharashtra-were-at-least-3-times-the-official-covid-toll/article35708965.ece Accessed August 3, 2021.

Rukmini S. Madhya Pradesh saw nearly three times more deaths than normal after second wave of Covid-19 struck. Scroll.In. June 12, 2021. Available from: https://scroll.in/article/996772/madhya-pradesh-saw-nearly-three-times-more-deaths-than-normal-after-second-wave-of-covid-19-struck Accessed June 12, 2021.

Rukmini S. Andhra Pradesh saw 400% increase in deaths in May, Tamil Nadu saw more modest excess mortality. Scroll.In. June 13, 2021. Available from: https://scroll.in/article/997427/andhra-pradesh-saw-400-increase-in-deaths-in-may-tamil-nadu-saw-more-modest-excess-mortality Accessed June 13, 2021. [“AP”]

Rukmini S. How India Could Fill In The Blanks On Excess Mortality. India Spend. June 17, 2020. Available from: https://www.indiaspend.com/how-india-could-fill-in-the-blanks-on-excess-mortality/ Accessed June 27, 2021.

Rukmini S. Daily Registered Deaths in Chennai. Chennai Municipal Corporation. Available from: https://github.com/elseasama/covid19chennai/tree/gh-pages/chennai_data Accessed September 7, 2021.

Saikia A. Assam saw 28,000 more deaths than normal in months when first wave of Covid-19 struck. Scroll.in. June 16, 2021. Available from: https://scroll.in/article/997683/assam-saw-28000-more-deaths-than-normal-in-months-when-first-wave-of-covid-19-struck Accessed June 17, 2021.

Scroll Staff. Gujarat is undercounting Covid-19 deaths, shows ‘Divya Bhaskar’ report. Scroll.In. May 14, 2021. Available from: https://scroll.in/latest/994906/gujarat-is-undercounting-covid-19-deaths-shows-divya-bhaskar-report Accessed June 6, 2021.

Srivatsa SS, Ramani S. Karnataka's excess deaths nearly 6 times official COVID-19 toll. The Hindu. June 20, 2021. Available from: https://www.thehindu.com/news/national/excess-deaths-in-karnataka-nearly-six-times-official-covid-19-tally/article34870624.ece Accessed June 21, 2021.

Srivatsa SS, Ramani S. Number of deaths registered in CRS system of Karnataka. June 21, 2021. Available from: https://docs.google.com/spreadsheets/d/1pBM4wFuGQgnL6syAG3IFHGUk9yzc4pToSv2tftKElxY/edit#gid=0 Accessed June 21, 2021.

Wall of Grief. The Reporters Collective, National Foundation of India. Gujarat Municipality Death Registers. September 2021. Available from: https://www.wallofgrief.org/ Accessed September 11, 2021.
